# The Role of Celiac Disease in Severity of Liver Disorders and Effect of a Gluten Free Diet on Diseases Improvement

**DOI:** 10.5812/hepatmon.11893

**Published:** 2013-10-13

**Authors:** Mohammad Rostami-Nejad, Thea Haldane, David AlDulaimi, Seyed Moayed Alavian, Mohammad Reza Zali, Kamran Rostami

**Affiliations:** 1 Department of Celiac Disease, Gastroenterology and Liver Diseases Research Center, Shahid Beheshti University of Medical Sciences, Tehran, IR Iran; 2 Department of Gastroenterology, Alexandra Hospital, Worcestershire, UK; 3 Baqiyatallah Research Center for Gastroenterology and Liver Diseases, Baqiyatallah University of Medical Sciences, Tehran, IR Iran; 4 Middle East Liver Disease Center, Tehran, IR Iran; 5 Department of Gastroenterology, Darent Valley Hospital, Darenth Wood Road, Dartford, UK

**Keywords:** Celiac Disease, Liver Disease, Severity

## Abstract

**Context:**

Celiac disease (CD) is defined as a permanent intolerance to ingested gluten. The intolerance to gluten results in immune-mediated damage of small intestine mucosa manifested by villous atrophy and crypt hyperplasia. These abnormalities resolve with initiationa gluten-free diet.

**Evidence Acquisition:**

PubMed, Ovid, and Google were searched for full text articles published between 1963 and 2012. The associated keywords were used, and papers described particularly the impact of celiac disease on severity of liver disorder were identified.

**Results:**

Recently evidence has emerged revealingthat celiac disease not only is associated with small intestine abnormalities and malabsorption, but is also a multisystem disorder affecting other systems outside gastrointestinal tract, including musculo-skeletal, cardiovascular and nervous systems. Some correlations have been assumed between celiac and liver diseases. In particular, celiac disease is associated with changes in liver biochemistry and linked to alter the prognosis of other disorders. This review will concentrate on the effect of celiac disease and gluten-free diets on the severity of liver disorders.

**Conclusions:**

Although GFD effect on the progression of CD associated liver diseases is not well defined, it seems that GFD improves liver function tests in patients with a hypertransaminasemia.

## 1. Context

Celiac disease (CD) or gluten sensitive enteropathy can be defined as a permanent intolerance to ingested gluten (a protein component stored in wheat, barley, and rye). Gluten intolerance results in immune-mediated damage to small intestine mucosa and induces villous atrophy and crypt hyperplasia (1, 2). These abnormalities improve with initiationa gluten-free diet (GFD).

Population studies from the United States have demonstrated that about 1:100 individuals were affected by CD (3). Clinical presentation of disorder can vary from a classic malabsorption syndrome to extra-intestinal symptoms such as infertility, iron deficiency anaemia, and osteoporosis. CD may also be presented subclinical and diagnosed unexpectedly on routine investigations foriron deficiency anaemia or symptoms of irritable bowel syndrome (1, 4, 5).

CD is associated with abnormal liver function tests. People with CD may also have liver conditions, such as primary biliary cirrhosis, autoimmune hepatitis, or primary sclerosing cholangitis (1). There is evidence that CD may modify clinical course of concurrent chronic liver diseases (1). In this review, we concentrated on CD effect on severity of liver disorders and also the impact of a GFD on diseases improvement.

## 2. Evidence Acquisition

### 2.1. Liver Involvement in Celiac Disease

Liver abnormalities in CD are common. Among patients who presented with typical symptoms of CD, liver blood test abnormalities have been reported in 40% of adults and 54% of children (6-8). In addition, CD is present in about 9% of patients presenting with a chronic unexplained hypertransaminasemia (9, 10).

CD may be associated with severe forms of liver disease (11). A population study in Sweden reported that individuals with CD were 2-6 times more likely to develop liver disease in later life compared to healthy controls. In addition, the study reported that patients known to have liver disease were 4-6 times more likely to develop CD compared to patients without liver disease (12). Patients with CD were also 8 times more likely to die from cirrhosis (13). Because of these findings, Green et al. (2002) suggested that CD should always be excluded before a diagnosis of cryptogenic cirrhosis is made (14) (see Table 1). 

**Table 1.** Associations Between Liver Disorders and Celiac Disease

Liver Disorders	Association, %	First Author and References
**Hypertransaminasemia**	9	Volta et al. (1998) (10)
**Hypertransaminasemia**	46	Bardella et al. (1995) (6)
**End-stage autoimmune liver disease**	3	Rubio-Tabia et al. (2008) (15)
**Autoimmune hepatitis**	4-6.4	Volta et al. (1998) and Villalta et al. (2005) (16, 17)
**Primary biliary cirrhosis **	0-11	Dickey et al. (1997), Kingham and Parker (1998), Gillet HR et al. (2000), Floreani et al. (2002), Volta U, et al. (2002), Bardella et al. (1997) (18-22)
**Sclerosing cholangitis**	1.6	Volta et al. (2002) (23)
**Non-cirrhotic intrahepatic portal hypertension (NCIPH)**	16	Eapen et al. (2011) (24)
**Hepatitis C**	1.2	Fine et al. (2001) (25)
**Chronic hepatitis C**	1.3	Durante-Mangoni E et al. (2004) (26)
**Hepatitis C**	No association	Rostami Nejad et al. (2010) (27)
**Hepatitis B**	10	Sima et al. 2010 (28)
**Hepatitis B**	No association	Leonadi and La Rosa (2010) (29)
**NAFLD/NASH**	3.5	Bardella et al (2004), Loiacono O et al (2005) (30, 31)
**Hemochromatosis**	Case reports and theoretical associations	Turcu et al. (2000), Heneghan et al. (2000), Butterworth et al. (2002), Barisani et al. (2004) (32-35)

## 3. Results

### 3.1. Hypertransaminasemia in Celiac Adult Patients

Bardella et al. (1995) investigated the prevalence of hypertransaminasemia in adults with CD and the effect of GFD in those patients. They evaluated 158 consecutive adult patients, 127 women and 31 men, aging 18-68 years (mean age: 32). At diagnosis, 67 patients (42%) experienced raised aspartate and/or alanine transaminase (AST and ALT, respectively) levels and 91 patients showed normal liver function tests (6). In order to compare patients with and without abnormal liver function tests, demographic data, body mass index, and severity of intestinal histological involvement were examined. Gluten-free diet was started for all patients and after 1 year, abnormal liver function tests improved significantly on a GFD (6).

In other several studies, it was demonstrated that a GFD has significantly reduced the degree of hypertransaminasemia in patients with CD. Normalization of serum transaminases was shown to occur in 75-95% patients, usually within a year of good adherence to the diet (6, 36). This reversible CD-related liver damage has been called celiac hepatitis (37).

### 3.2. Autoimmune Liver Disease

The association of CD with type 1 and type 2 autoimmune hepatitis was shown in studies by Villalta et al. and Volta et al. (16, 17). In both studies, the diagnosis of CD was made histologically and in either study, very few patients demonstrated classical symptoms of CD (16, 17). The observed relationship between CD and autoimmune liver disease is thought to occur as a result of linkage disequilibrium. Both CD and autoimmune hepatitis are associated with specific class II HLA molecules encoding for HLA complex genes on chromosome 6 (38). The effects of a GFD on natural history of autoimmune hepatitis are not clear but a GFD is necessary to improve any symptoms due to CD (39, 40).

In 2008 Rubio-Tabia et al. published the results of a study investigating associations between CD and autoimmune liver diseases, primary biliary cirrhosis, primary sclerosing cholangitis, and autoimmune hepatitis (15). In this study, the prevalence of tissue transglutaminase antibodies (tTGAs) and endomysial antibodies (EMAs) in end-stage autoimmune liver disease (ESALD) were reported (15). Levels of tTGA were measured in blood samples from 448 patients prior to liver transplantation out of which 310 patients showed ESALD and 178 patients a non-autoimmune disease. Positive samples were tested for EMA sand re-checked at 6, 12, and 24 months intervals after transplantation (15). The study reported that 3% of ESALD patients showed evidence of CD compared to 0.6% of those with non-autoimmune disease, demonstrating a fivefold greater risk of CD in those with ESALD.

After transplantation, tTGAs and EMAs were normalized in 94% and 100% of patients, respectively, without gluten elimination. Also, 3 out of 5 patients with classical symptoms of CD showed a symptomatic improvement. Two cases of intestinal lymphoma were reported in two patients with no clinical manifestations of CD. In patients with ESALD, both tTGA and EMA levels decreased following liver transplantations without gluten withdrawal. The authors concluded that the symptoms of CD may have been improved by a post-transplantation immune suppression but such improvements may not prevent the disease from progressing to intestinal lymphoma. The study did not provide any insight into the whether early detection and treatment of CD would influence the identified risk of developing ESALD, if liver disease contributed to the risk of developing CD, or if some third unknown connection linked the ESALD and CD (15).

### 3.3. Primary Sclerosing Cholangitis

Volta et al. (2002) conducted a study on 61 patients with primary sclerosing cholangitis (PSC) and found the prevalence of CD as 1.6% (22). In addition, a large study on general population in Sweden suggested that the prevalence of PSC in patients with CD was increased by 4-8 folds, compared to the general population (12). This study was dependent upon hospital discharge data. Furthermore, as CD is a common disorder, the possibility of this relationship occurring by chance could not be excluded (12).PSC is strongly associated with ulcerative colitis (UC). Interestingly, tTG antibodies have been shown to be present in sera and faecal supernatants of patients with UC, and that the antibody concentrations correlated with the disease activity (23). However, there is no evidence of a true association between UC and CD. It has been suggested that further studies would be required to investigate relationship between CD and PSC (1).

### 3.4. Primary Biliary Cirrhosis

An association between Primary Biliary Cirrhosis (PBC) and CD was first described in 1978 (41). Since then, this association was studied by screening patients with PBC for CD. The reported prevalence of CD in PBC patients varies considerably from 0% to 11% in different studies (18-22, 42). There have also been two large population-based studies supporting an association between CD and PBC (43, 44).

There are several common pathophysiological processes occur in CD and PBC, including increased intestinal permeability (demonstrated in both conditions) (45), an increase in gut-derived antigens in the portal system of patients with either CD or PBC, compared to controls (46); and possibly shared genetic susceptibility to PBC and CD (47).

Dickey et al. (1997) demonstrated that abnormal liver tests did not improve in patients with subclinical CD and PBC after receiving a GFD for 24 months despite the disappearance of endomysial antibody in the serum. This suggests that either adherence to a gluten-free diet is unable to reverse pathological process in PBC, or that PBC and CD share common etiological factor(s) but pathologically with independent processes (19).

### 3.5. Non-Cirrhotic Intrahepatic and Idiopathic Portal Hypertension (NCIPH and IPH)

Non-cirrhotic intrahepatic portal hypertension (NCIPH) is portal hypertension occurs within the liver not being triggered by cirrhosis. NCIPH is generally regarded as a benign process (24). Eapen et al. (2010) investigated whether or not gut-derived prothrombotic factors may contribute the pathogenesis and prognosis of portal hypertension. In this study a cohort of patients were followed, prognostic indicators were analyzed gut associated disorders were looked in 34 patients. In this study, five out of 31 patients showed CD and presence of CD was a predictor of shorter transplant-free survival for these patients. The study also showed that liver failure develops edultimately in 53% of NCIPH patients. There were no patients with CD in the study without concomitant liver disease. The authors concluded that intestinal disease would play a significant role in the pathogenesis of intrahepatic portal vein occlusion leading to NCIPH (24).

Idiopathic portal hypertension (IPH) is clinically categorized by obvious splenomegaly, pancytopenia, portal hypertension, and relatively mild anomalies in liver function tests with unknown etiology (48, 49). In 2009, Zamani et al. reported a case study from a 54-year-old man who was admitted for evaluation of depression, weight loss, abdominal pain, and lower limb edema (50). The patient showed pancytopenia with large volume ascites, splenomegaly, and esophageal varices consistent with portal hypertension. His serology and duodenal biopsy confirmed CD. The symptoms improved when a gluten-free diet was initiated. However, his clinical course was complicated later by ulcerative jejunoileitis and intestinal T-cell lymphoma. The authors suggested that idiopathic portal hypertension could develop in patients with CD that was caused by an increasing immune reaction in the spleno-portal axis.

### 3.6. Viral Hepatitis

It has been suggested that infection with Hepatitis B and Hepatitis C viruses may trigger immunologic intolerance to gluten (48, 49). There is no evidence of an association between hepatitis B or hepatitis C infections and CD (48).

There have been few studies investigating an association between hepatitis C infection and CD. Fine et al. (2001) identified 259 cases with chronic hepatitis C disease in which CD was found by three times more compared to control group (1.2% versus 0.4% ). The diagnosis of CD was confirmed by performing a duodenal biopsy (25). Durante Mangoni et al. (2004) showed the prevalence of celiac disease among 534 patients with chronic hepatitis C as 1.3% (26). In a cross-sectional study by Rostami Nejad et al. (2010), 827 pregnant women were serologically screened for CD and HCV antibodies; the result of this study suggested no correlation between two disorders (27).

Importantly, CD may present for the first time during treatment of hepatitis C infection, as ribavirin and interferon Alfa enhance type 1 T helper cell immune responses and increase interferon gamma gene expression (51-56). Therefore, CD should be considered in patients who develop unexplained diarrhea during treatment of hepatitis C infection.

In a study by Sima et al. (2010) on 88 patients with chronic hepatitis B, 10% were serologically positive for celiac autoantibodies (28). Leonadi and Rosa (2010) examined 60 chronic carriers or infected patients who were recovered from hepatitis B infection by performing celiac serology. Some of the carriers were treated with Alfa interferon over a year among them no IgA deficiency was present. In all 60 patients, both IgA anti-endomysial antibodies (EMA) and IgA transglutaminase antibodies (tTGA) were negative implying no association between hepatitis B infection and CD (29).

Lastly, patients with CD showed high rates of non-response to hepatitis B vaccine (48, 57-61). Non-response rates have been reported as high as 54% in children with CD and 68% in adults with CD (62, 63). It has been speculated that the inadequate response to vaccination may be related to HLA DQ2 haplotype molecule (62). However, many recent studies suggest that a lapse in GFD is the most common cause of low response to vaccination in CD (64).

### 3.7. NAFLD

Approximately 10-25% of general population will develop non-alcoholic fatty liver disease (NAFLD) (65, 66). The body mass index of 27% of Americans whom recently been diagnosed as CD patients was > 25, demonstrating them as overweight or obese (39). 

Some studies have shown that intestinal permeability may play a role in the pathogenesis of NAFLD, which is hepatic component of metabolic syndrome. NAFLD can be degraded into non-alcoholic steatohepatitis for which liver exposure to gut bacteria was hypothesized (30, 31).

Miele et al. (2009), compared the results of gut permeability in a group of patients with NAFLD with patients suffering from untreated CD and healthy volunteers (67). Thirty-five NAFLD patients proved by biopsy, 27 patients with CD, and 24 healthy volunteers were evaluated. Levels of small intestine bacterial overgrowth were measured in all patients by a glucose breath test, and intestinal permeability was assessed by urinary excretion of Cr-EDTA. Integrity of tight junctions within the gut was analyzed by duodenal biopsies. The study mainly found that intestinal permeability and prevalence of small intestine bacterial overgrowth were increased in patients with NAFLD correlating with severity of steatosis. Disruption of tight junction integrity may explain the increased permeability in these patients (67). Abnormalities in liver function tests were noted in patients with NAFLD and CD. These abnormalities were shown to resolve after 6 months of taking a GFD (30, 31). There was no evidence on reverse of histological abnormalities affecting liver (30, 31).

### 3.8. Hemochromatosis

Hemochromatosis and CD are associated through two ways. Case reports have shown that iron overload and diagnosis of hereditary hemochromatosis often follows successful celiac treatment (34, 35). British patients with CD have a high incidence of mutations in hemochromatosis gene (HFE) (34). This may indicate that enhanced iron production is an adaption to reduced nutrient absorption associated with CD (1, 33). However, a study of Italian CD patients showed no increased incidence of HFE mutations in patients with CD and therefore, the association between hemochromatosis and CD might be a chance finding (1, 32).

### 3.9. Liver Transplantation

A research group from Finland reported from four patients with severe liver diseases concomitant with CD. One patient suffered from congenital liver fibrosis, one from massive hepatic steatosis, and two from progressive hepatitis without an apparent cause. Three out of four individuals were considered for liver transplantation but a GFD reversed hepatic dysfunction in each case (68). Another study investigated patients undergoing liver transplantation. Of 185 patients who underwent transplantation, 8 (4.3%), (over four times the normal population) patients were positive for CD. The diagnosis of CD was made in 6 out of 8 patients before transplantation, one of which was already on a GFD. Of these eight individuals, three patients were diagnosed as PBC, one as autoimmune hepatitis, one as PSC, one as congenital liver fibrosis, and one assecondary sclerosing cholangitis. Not all the patients showed symptoms of CD. The authors suggested: “gluten dependence immunologically induced extra intestinal manifestation of celiac disease”, and that patients with severe liver disease should be investigated for CD (68).

### 3.10. Liver Inflammation and Fibrosis

Transglutaminases are a group of calcium-dependent enzymes playing role in functions such as wound healing, repair of damaged tissue, fibrogenesis, apoptosis, inflammation, and management of cell cycle. This implies that they have a key role in autoimmune, inflammatory, and degenerative diseases. A recent report highlighted present conception of transglutaminase function in gastrointestinal and liver diseases (69). Transglutaminase-2 is of central importance, as it is crucial in CD pathogenesis and influences inflammation and fibrogenesis in inflammatory bowel as well as chronic liver diseases, suggesting a target role for treatments in the future (69)

### 3.11. The Pathogenesis of Liver Disease in Celiac Disease

It is not known why damage to the liver occurs in CD. Patients with CD exhibit damaged gut mucosa and increased gut permeability (46). Gluten toxicity and intestinal permeability have been proposed as factors. Schwabe et al. (2006) suggested that the pathogenesis may contain an immune reaction involving “toll-like receptors” (46). “Toll-like receptors” (TLRs) are present on many surface cells controlling and mediating immune functions. TLRs sense molecules present on the pathogens, but not present on the host cells; thereby, cytokines are released setting off inflammatory and anti-pathogen responses. Lipopolysaccharides (LPS) are a class of molecules recognized by TLRs, which are common in most pathogenic bacteria. Gluten increases intestinal permeability, disrupting intestinal barrier in small intestine of patients with CD. This disruption may permit endotoxins from gut bacteria such as LPS to reach liver portal vein and trigger a TLR-mediated inflammatory response from immune cells within the liver, leading to the further release of pro-inflammatory mediators and ultimately to inflammation and liver damage in CD. It is thought that gluten -by itself could also trigger a liver immune response. Kupffer cells in the liver are capable of antigen presentation to T- cells, along with liver dendritic cells, which may initiate a T-cell response to gluten within the liver (46).

Malabsorption, increased intestinal permeability, bacterial overgrowth, malnutrition, and chronic intestinal inflammation have all also been suggested as possible mechanisms for liver injury formation in CD.

## 4. Conclusions

In this paper we have outlined important evidence in respect to CD and liver disorders. The abnormalities in liver function associated with CD, and evidence for association between CD and various liver diseases have been discussed. It seems that there are stronger links between CD and autoimmune liver disorders. There is a need for more researches to demonstrate whether or not CD is associated with primary sclerosing cholangitis. CD seems to be related to contributing portal hypertension where no liver disease is evident. There are some associations between CD and response to Hepatitis B vaccine. Treatment of hepatitis C infection seems to precipitate CD in some patients. There are links between NASH/NAFLD and CD.

**Figure 1.**
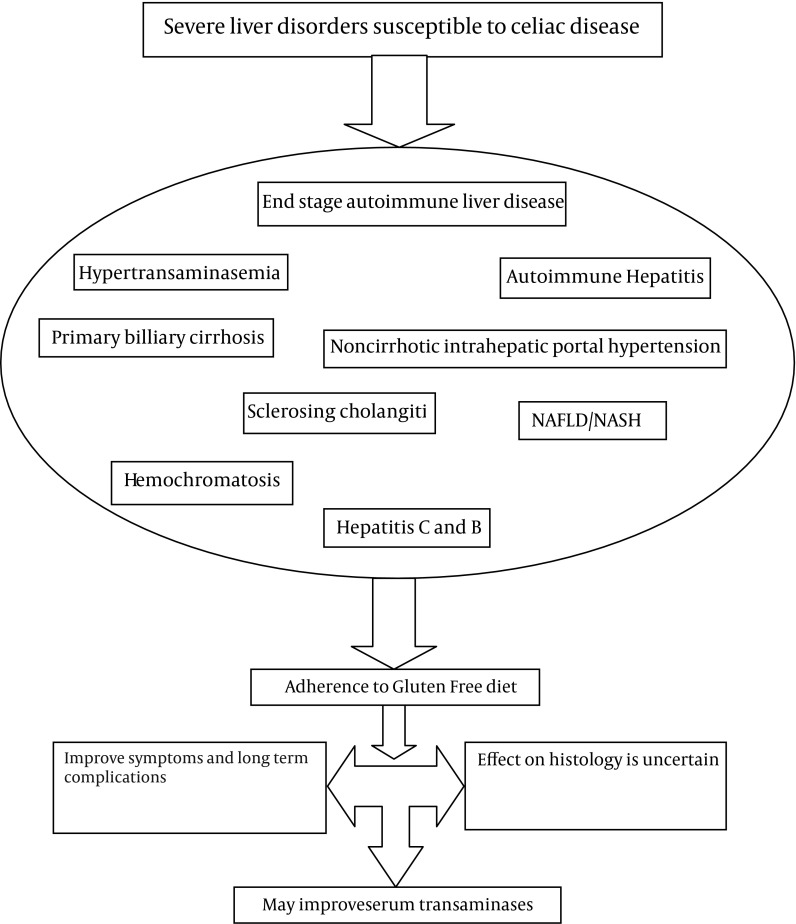
Effect of GFD in Patients With Severe Liver Disorders

The Pathogenesis underlying liver damage in CD is not fully defined but several mechanisms have been suggested. Figure 1 summarizes articles discussed in this paper. 

The liver diseases associated with CD can be severe, and therefore, CD should be considered in a patient presenting with hepatic failure. Liver abnormalities ranging from mild alterations in transaminases to hepatic failure in the setting of CD can be treated with a gluten free diet (6, 36). It is not clear what the effects of a gluten-free diet are on the severity of other liver diseases, for example, autoimmune hepatitis. Regardless of the effect of a GFD on a coexisting liver disorder, a GFD is necessary to improve symptoms of celiac disease and any long-term sequelae (39, 40).
